# Cytodiagnosis of glomus tumor

**DOI:** 10.4103/0970-9371.71876

**Published:** 2010-07

**Authors:** Sumana Mukherjee, Gautam Bandyopadhyay, Sandeep Saha, Manoj Choudhuri

**Affiliations:** Department of Pathology, Bankura Sammilani Medical College, West Bengal, India

**Keywords:** Fine needle aspiration cytology, glomus tumor, subungal

## Abstract

Glomus tumors are uncommon, with an estimated incidence of 1.6%. Cytological descriptions of this tumor are few. We report a 15-year-old boy presenting with a painful subungual swelling. Fine needle aspiration cytology showed uniform cells with homogeneous chromatin and scanty cytoplasm. Cytology was reported as “suggestive of glomus tumor”. Histopathological examination confirmed the diagnosis. Careful cytomorphological examination supported by appropriate clinical history should suggest the diagnosis of glomus tumor and help in preoperative diagnosis.

## Introduction

Glomus tumor is a distinctive neoplasm, the cells of which resemble the modified smooth muscle cells of the normal glomus body. The normal glomus body is a specialized form of arteriovenous anastomosis that serves in thermal regulation. It is located in the stratum reticularis of the dermis and is most frequently encountered in the subungual region, the lateral areas of the digits and the palm. The tumors are uncommon, with an estimated incidence of 1.6%.[1] We present a case of a 15-year-old boy with a glomus tumor in the subungual region diagnosed by cytology. As the tumors occur rarely, cytomorphological descriptions are few.[2–4]

## Case History

A 15-year-old boy presented with a painful swelling in the subungual region of the right index finger, about 1.5 cm in diameter. Radiographs demonstrated a soft tissue lesion with no bony involvement. Fine needle aspiration cytology of the swelling was performed.

**Figure 1:**
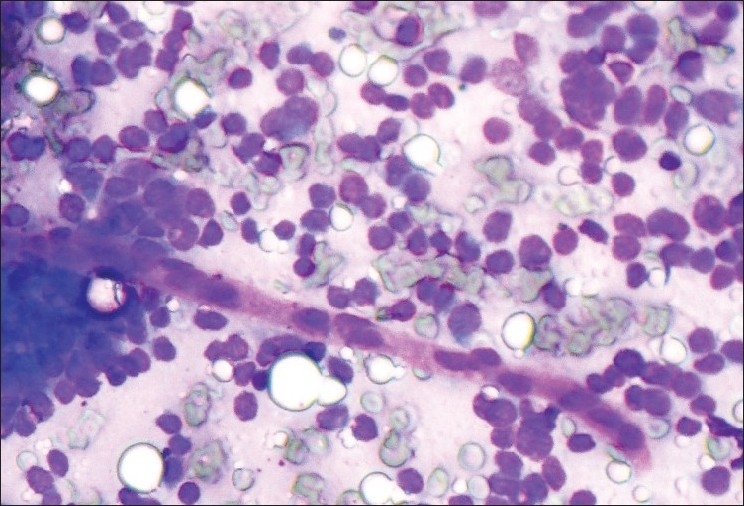
Round to oval cells with homogenous granular chromatin, scanty cytoplasm and indistinct cell borders. A capillary crossing a cell cluster (MGG, ×400)

Smears exhibited groups of cohesive, uniform, small, round to polygonal cells with scanty cytoplasm, indistinct cell borders and round nucleus with homogeneous chromatin. In few cell clusters, very scanty, wispy intercellular myxoid material was seen. Occasional capillaries were seen crossing cell clusters. Cytology was reported as “suggestive of glomus tumor” [Figure 1].

Surgical excision was followed by histopathological examination. Histologically, the lesion was well circumscribed, consisting of capillary- sized vessels surrounded by collars of glomus cells with a rounded, regular shape and a punched-out regular nucleus set off from the amphophilic cytoplasm. The outlines of the cells were not well defined. Reticulin stain confirmed the morphology of glomus. A histopathological diagnosis of glomus tumor was made.

## Discussion

The first cytological description of glomus was given by Holck *et al*.[4] in an axillary mass misdiagnosed as ectopic breast tissue.

Glomus tumors cause little diagnostic difficulty at histopathology, especially if the clinical presentation is typical. However, glomus can also occur in the gastrointestinal tract, solid organs (liver, kidney) and the extremities.[5] There is a recent report of a glomus in the stomach diagnosed by endoscopic ultrasound-guided fine needle aspiration cytology.[3] Cytomorphological characterization of a classical case of glomus tumor can help in cytological diagnosis at uncommon sites. Cytomorphologic features have been poorly defined. Reports have described cohesive clusters of uniform round cells with scanty cytoplasm, similar to the present case.[3,6,7] Gu *et al*.[7] have described a background of scattered amorphous material similar to the wispy magenta material in our case. Debol *et al*.[3] have described a background with vascular channels and Vinette-Leduc *et al*.[6] have found a background of blood, bare nuclei and occasional inflammatory cells.

One of the difficulties at aspiration could be a hemorrhagic aspirate. Paucicellularity was reported by some authors.[3,7] The authors suggest needling of the tumor without aspiration. The differential diagnoses are many. Eccrine spiradenoma may present a difficult diagnostic problem.[8] However, the localization of glomus cells around blood vessels and lack of acini formations are helpful features.[9] Smears of eccrine spiradenoma show the presence of bland uniform cells in cohesive clusters and cribriform sheets with rosette-like structures surrounding the amorphous material. Cytologic distinction rests on identifying three types of cells – larger epithelial cells, myoepithellial cells and smaller lymphocytes.[8] Glomus tumors have to be differentiated from other vascular lesions, such as hemangiopericytoma, paraganglioma and lobular hemangioma, depending on the site of origin of the tumor. In hemangiopericytoma, cellular smears show knobby clusters of oval to spindle-shaped cells with ill-defined, finely granular cytoplasm and bland nuclei, but the number of mitotic figures varies. In paragangliomas, cells may show moderate nuclear pleomorphism with fine red granules in the cytoplasm. Lobular capillary hemangioma show clusters of oval to spindle-shaped cells along with a cellular infiltrate of neutrophils and mononuclear cells.[2] Because glomus tumor is derived from pericytes with special modification toward glomus cells, it is closely related to myopericytoma and myofibroma. In general, the glomus tumor consists of more rounded cells related to blood vessels whereas the other two lesions tend to have larger, less-rounded cells with more cytoplasm and ill-defined cell borders. However, glomus tumor with spindle cell morphology might exhibit overlapping cytologic features.[10] Tumors located in the gastrointestinal tract should be differentiated from gastrointestinal stromal tumors. For all differential diagnoses, careful examination of cytological features described, like characteristic chromatin, indistinct cytoplasmic borders and presence of few vessels, should be helpful.

Accurate clinical history and cytology can diagnose glomus tumors in most cases.
